# The Unique Cysteine Knot Regulates the Pleotropic Hormone Leptin

**DOI:** 10.1371/journal.pone.0045654

**Published:** 2012-09-24

**Authors:** Ellinor Haglund, Joanna I. Sułkowska, Zhao He, Gen-Sheng Feng, Patricia A. Jennings, José N. Onuchic

**Affiliations:** 1 Department of Chemistry and Biochemistry and Center for theoretical Biological Physics (CTBP), University of California San Diego, La Jolla, California, United States of America; 2 Department of Pathology; School of Medicine and Molecular Biology Section, Division of Biological Sciences, University of California San Diego, La Jolla, California, United States of America; 3 Center for Theoretical Biological physics and Department of Physics and Astronomy, Chemistry, and Biochemistry and Cell Biology, Rice University, Houston, Texas, United States of America; Fundação Oswaldo Cruz, Brazil

## Abstract

Leptin plays a key role in regulating energy intake/expenditure, metabolism and hypertension. It folds into a four-helix bundle that binds to the extracellular receptor to initiate signaling. Our work on leptin revealed a hidden complexity in the formation of a previously un-described, cysteine-knotted topology in leptin. We hypothesized that this unique topology could offer new mechanisms in regulating the protein activity. A combination of *in silico* simulation and *in vitro* experiments was used to probe the role of the knotted topology introduced by the disulphide-bridge on leptin folding and function. Our results surprisingly show that the free energy landscape is conserved between knotted and unknotted protein, however the additional complexity added by the knot formation is structurally important. Native state analyses led to the discovery that the disulphide-bond plays an important role in receptor binding and thus mediate biological activity by local motions on distal receptor-binding sites, far removed from the disulphide-bridge. Thus, the disulphide-bridge appears to function as a point of tension that allows dissipation of stress at a distance in leptin.

## Introduction

A single mutation found in the leptin gene led to the discovery of the role of this protein in regulating obesity [1]. While the cases of mutation associated morbid obesity constitute an orphan family of disease targets [2], [3], [4], [5], [6], leptin is now recognized as an essential factor in signaling from adipose tissue to the brain and regulates the propensity towards developing diabetes [7]. In addition, leptin is a pleiotropic hormone involved in the regulation of inflammation, hematopoiesis as well as a major regulator of the innate and adaptive immune response [8]. Importantly, a number of mutations in the human gene have been linked to several developmental processes and diseases including Alzheimer's, and puberty onset as well as diabetes [9], [10], [11], [12]. Only one of these mutations maps directly to the receptor-binding site [2], [3], [13] and some far from this site have been suggested to be linked to the formation of a single disulphide bond in the protein [4], [5], [6]. The four long helices in leptin form a helical-bundle motif containing one disulphide bridge (PDB code 1AX8 [14]). The literature suggests that helical-bundles are quite stable proteins (8–15 kcal/mol [15], [16], [17], [18]). Our work shows that the stability of leptin is much lower then seen with the typical helical-bundles (1.8^Reduced^ *versus* 3.4^Oxidized^ kcal/mol, respectively). We analyzed the structure and found that the formation of the disulphide bond not only stabilizes the protein but also leads to a previously un-described, uniquely cysteine knotted structure in the oxidized state. The question then arises: why does leptin introduce this unique topology when other four-helix-bundles are stable enough to exist without a knot (between 8.3–14.5 kcal/mol [15], [17]) or a disulphide bridge (9.4 kcal/mol [18]). The discovery of the previously uncharacterized structure led us to hypothesize that the unique knotted-topology could offer new mechanisms of regulation in leptin. Thus, disease mutations may be linked to formation of the cysteine knot structure rather than simply affecting the disulphide bond. Additionally, it is not known if this knotted topology can spontaneously fold from the unfolded to the native basin or spontaneously self-tie/untie. If the protein can solve this obstacle on the folding pathway the remaining question is: how different are the rate constants for the oxidized and reduced route?

**Figure 1:**
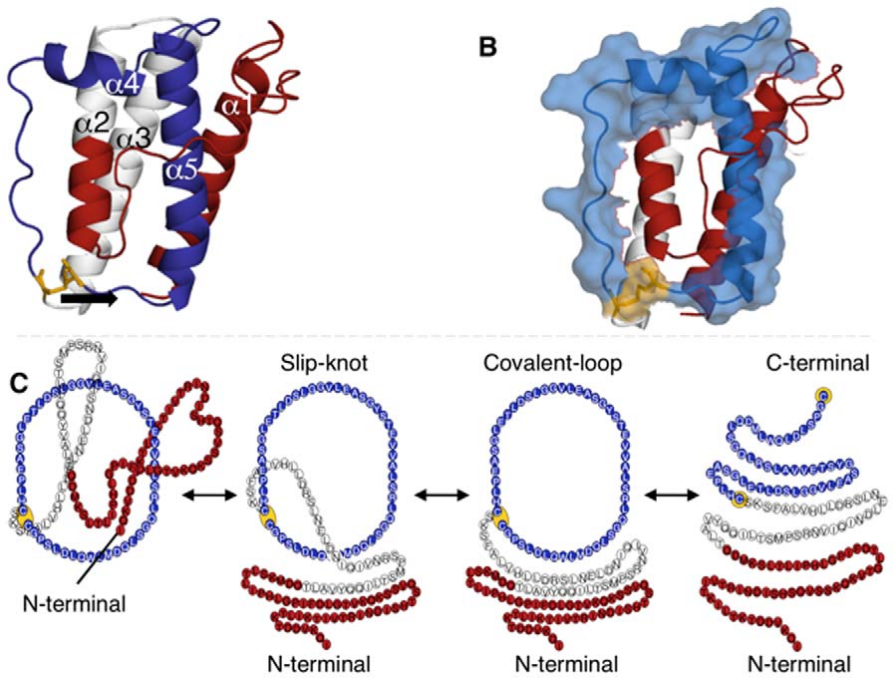
The unique cysteine knotted helical bundle of leptin. *(A)* The native structure of leptin (PDB code 1AX8 [14]). The cysteine bridge is located between residue C96 and the C-terminal cysteine (yellow), indicated with an arrow. The overall conformation of the protein creates a cysteine knotted helical bundle (blue). Consequently, 59 of the N-terminal residues (red), helix 3 and half of helix 2 (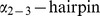), have to be ‘threaded’ through the covalent-loop (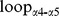). *(B)* The same structure as in *(A)*, but the covalent-loop is represented as Van der Waals spheres. The spheres illustrate the occupation of the side groups in the interior of the covalent-loop, showing that the side groups mainly point away from the center of the loop. In consequence, creating more open space to thread the 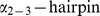. *(C)* A cartoon representation of leptin, native- (N), slipknotted (S), denatured- (D), and unfolded (U) conformation seen from left to right respectively. The denatured and unfolded state shows the starting conformation from which successful folding can proceed. The native state (*left panel*) show that leptin can reach an unfolded state, through a slipknotted conformation, without breaking the disulphide bond, thus leaving the covalent-loop intact in a ‘so-called’ denatured state. To reach the fully unfolded state linear state, (*right panel*) reduction of the disulphide bond is required.

Investigating the role of mutations in mediating leptin related diseases requires the understanding of the folding of the cysteine knotted motif and the formation of the fully active protein. In Figure 1 we show the overall topology of leptin emphasizing the role of the disulphide bond in the formation of the threaded structure. Under oxidized conditions, the C-terminal cysteine forms a disulphide bond to C96 forming a covalent-loop. The more commonly referred class of proteins with a knotted topology is the classic cysteine knotted proteins, such as the ICK motif, the conotoxins and the cyclotides [19], [20]. Here the cysteine knots are built upon a ß-structure with the disulphide bridges building the knot. In the case of leptin, the protein forms a cysteine knotted four-helix bundle, which differs from the common fold.

Our approach to understanding the role of mutations in regulating activity has been to investigate the free energy landscape and function of the protein of interest. The unique topology of leptin could be compared to slipknotted proteins [21], [22], which are of similar size. A slipknot topology appears when one loop is partially threaded across the other loop (Figure 1C) [23]. The knotted motif revealed in leptin represents the simplest slipknotted topology. Recent theoretical results show that the concept of a funneled landscape is applicable for slipknotted proteins [24]. However, these results have not been verified experimentally. In this work we can, for the first time, characterize the unique topology of leptin and theoretically explore the mechanism of reversible threading of a helical hairpin across the covalently bonded loop. We investigate the folding mechanism of the linear and knotted protein and compare the folding routes. The bifurcation on the protein landscape is discussed on a kinetic level. The differences in the rate limiting steps between the two topologies are investigated. We explore the motion of the native state of leptin and discovered a correlation between the binding site of leptin and the knotted topology. Finally, we demonstrate that leptin can successfully fold and unfold in both its oxidized and reduced state *in vitro*. Both these states activate the leptin receptor in human cell lines, suggesting that leptin can spontaneously fold and unfold from both states as shown by our theoretical investigation. Moreover, we suggest that the knot formation and the slipknotted topology are important not only in signaling from adipose tissue but also in host defense response in proteins such as cytokines and interferons [25], [26], [27].

## Results and Discussion

Leptin is a potential therapeutic target for regulating the common occurrence of ‘yoyo cycle’ of weight loss and weight gain for people that struggle with metabolic syndrome as a result of mutation in the leptin gene. Therefore, a means to produce active native protein is an emerging area of research in treating patients that have leptin deficiency.

The unique topology of leptin provides a considerable barrier to produce high yields of active protein from inclusion bodies. We sought to undertake this challenging problem in protein folding and additionally found a surprising correlation between the knotted topology and the function/activity of protein. As described in the introduction and Figure 1 leptin has a unique, slipknotted structure created by a disulphide bridge between the C-terminal and residue 96 to make a 50 residue long covalent-loop. Theoretical and experimental work indicates that proteins are able to solve/bypass these topological traps on the folding landscape [23], [24], [28]. However, the final question is how leptin can fold successfully, without any external help (chaperons), under the given topological constraint. This topology necessitates threading of the N-terminal across the covalent-loop (Figure 1). The significance of the disulphide bridge is debated from an experimental point of view [29], [30], [31], [32], [33]. It has been suggested that leptin requires the correct disulphide formation to fold [29], [30], [33]. However, the work of Imagawa *et al.* [31] implies that the protein can fold under reduced conditions [31]. To characterize the funnel landscape for proteins with a cysteine knotted conformation we apply numerical *in silico* simulations followed by in vitro and activity assays in human cell lines.

### Topology of the Protein

We found that leptin constitutes a new structural motif within the helical bundle family (PDB code 1AX8 [14]). The mature form of leptin has 146 residues (16 kDa) and its structure contains four antiparallel α-helices (a four helix bundle formed by α1, α2, α3 and α5) plus a short helix/turn (α4), packing almost perpendicular to the four-helix bundle (Figure 1A) [1]. There are two cysteine residues in leptin, the C-terminal (C146) plus C96, that can form a disulphide bridge. This generates a 50 residue covalent-loop (a tadpole like structure [34], Figure 1) that encompasses α4 and α5. We call this a covalent 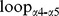 and an open 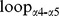 in the oxidized and reduced state respectively. This disulfide bond formation is thought to be essential for receptor signaling activity by maintaining the 36° kink in helix 5 [1]. Our re-examination of the structural coordinates reveals that disulphide-linked leptin constitutes a new member of the growing motifs of cysteine knotted proteins. However, the structure of leptin differs from the traditional cysteine knotted fold [35], [36], [37]. We term this new structure a ‘cysteine knotted helical bundle’. In brief, in the native state helices 2 and 3 form a helical hairpin motif (which we call the 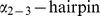 motif throughout this paper) that threads through the 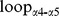 (Figure 1A). It is important to point out that this slipknotted structure differs from the typically observed loop-crossings seen in other slipknotted proteins as the 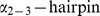 only crosses the 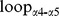 once in leptin (Figure 1). Additionally, leptin is composed of the simplest knot [38] as opposite to the more complex topologies like the trefoil knots [39]. Proteins with a similar topology are the lasso peptides [40], where the C-terminal tail is threaded through and caught within an N-terminal macrolactam ring. While these proteins share the same topology, they are very small peptides (16–21 residues) with less then 5 residues threaded through the closed-loop. Comparing the structures also reveal that the loop in the lasso peptides are enclosed by a glycine to glutamate contact, instead of a cysteine bridge, as seen in leptin. Oxidized leptin unfolds leaving the covalent 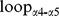 intact, held together by the disulphide bridge. However, reducing conditions breaks the bond to form a linear chain (unknotted) (Figure 1C). The disulphide bridge is conserved throughout the leptin family [14], [41], [42], suggesting that the unique cysteine knotted motif is conserved (Figure S1). This motif distinguishes leptin from the typical helical bundle cytokines, and could complicate and challenge the folding route significantly.

### Native state dynamics and receptor signaling

**Figure 2:**
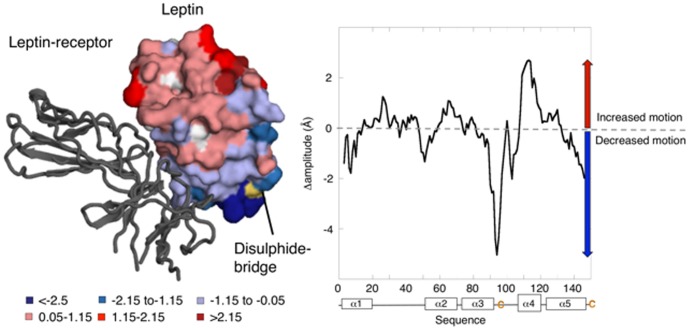
The crystal structure of leptin modeled onto its receptor showing the native state dynamics in the reduced and oxidized state. The results of the dynamic simulations of leptin (PDB code 1AX8) are mapped on to the structure of the receptor complex (PDB code 3V6O). Our analysis of the native state dynamics reveal how the formation of the disulphide bridge changes the native state dynamics from increased motions shown in red to decreased motions shown in blue. The disulphide bridge, indicated in yellow, clearly changes the overall motions in leptin. The plot shows the differences between oxidized and reduced protein where the positive numbers indicates regions with higher dynamics in the oxidized state, while negative numbers indicate higher motions in the reduced state.

Investigation of native state dynamic and frustration in proteins led to the discovery that dynamic regions in proteins are essential to protein function [43], [44]. It has also been proposed that frustrated surface regions are sites relevant for allostery [45]. To characterize the native state dynamics of leptin we performed All-Atom structure based simulations (Figure 2) and essential dynamics [46] of the first four eigenvectors of the 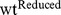 and 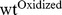 form of leptin. It was shown previously that protein-protein recognition [47], protein-DNA binding sites [48], enzyme-substrate binding and enzyme activity [49], [50] are all determined, partially, by conformational flexibility of the protein chain. We found significant differences in the amplitude and position of motions of individual amino acids along the sequence. The results reveal that the disulphide bridge has a more important role then previously known in leptin. It seems that the added constraint in the oxidized state changes the dynamics in distal receptor-binding sites (shown in red in the structure in Figure 2), far removed from the disulphide bridge. This suggests that the disulphide bridge provides a mechanism as a point of tension that dissipates stress across the motif of leptin. Reducing the disulphide bridge reduces the dynamics around the receptor binding sites in α1 and α2.

### The Free Energy Landscape

We define the reaction coordinate(s) RMSD, measuring the similarity between native and denatured structures, and Q, as the fraction of native contacts formed at any point, along the free energy landscape. This approach allows for a qualitative and quantitative interpretation of folding data [51], [52], [53], [54], [55]. However, oxidizing conditions creates an unusual geometric constraint in both the native and denatured states of leptin, as described in Figure 1.

**Figure 3:**
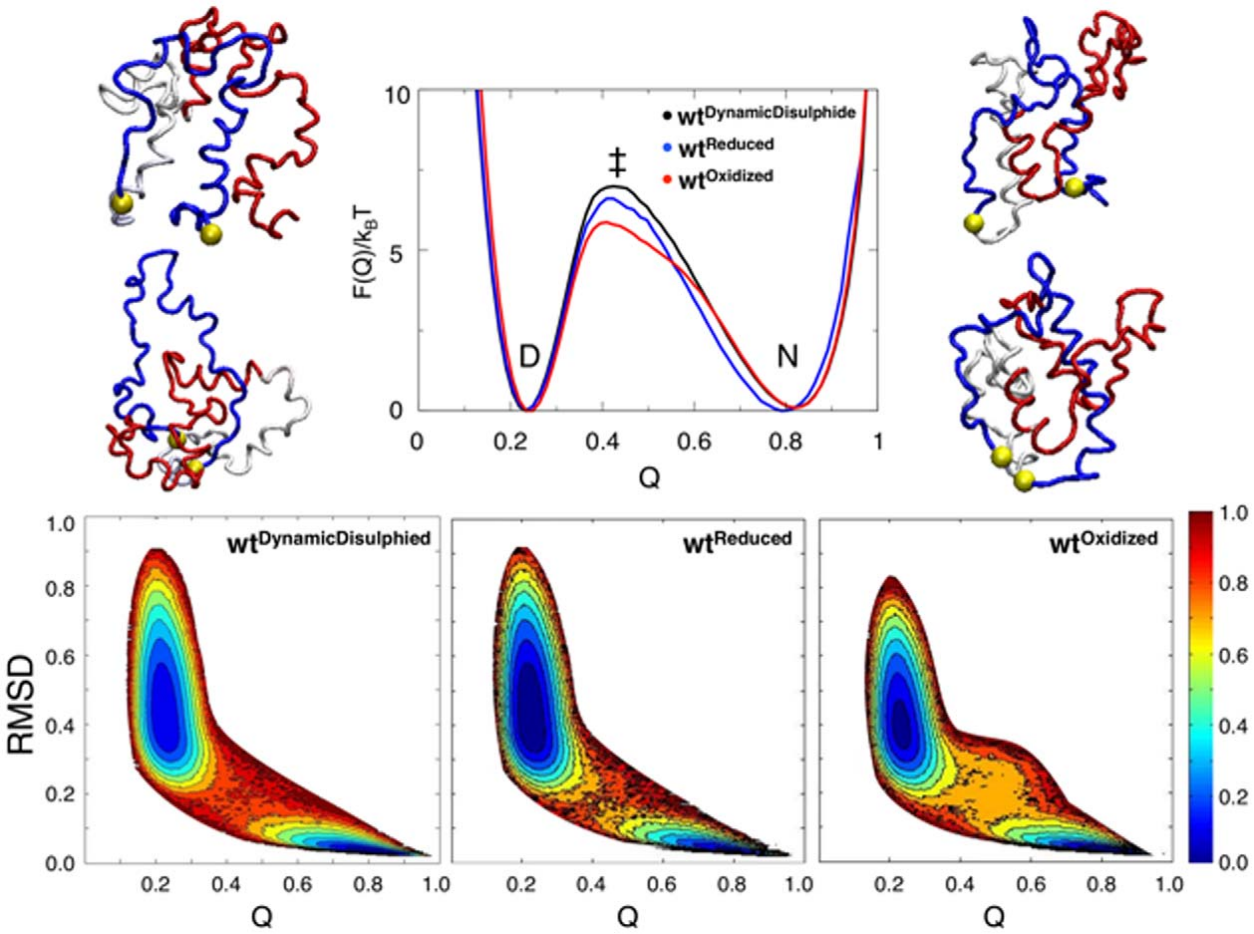
The free energy plot and representative structure diversity of leptin. *Top panel*: The free energy landscape for three folding routes of leptin, 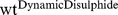 (black), 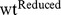 (blue) and 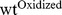 (red). It is clear from the folding trajectories that the 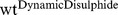 model can follow both the oxidized and the reduced routes. Therefore, four structures on the folding route are shown, two structures before the TS (Q = 0.3) and two at TS (Q = 0.4), on the left and right side of the free energy landscape respectively. The structures represented before the TS show that the 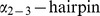 is structured and with the 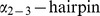 in a native conformation while the rest of the structure is denatured. At the TS most of the protein is folded, only *α*1 is unfolded. *Bottom panel:* The three dimensional representation of the distribution of structure diversity, measured by Q versus RMSD (F(Q, RMSD) where colors indicate normalized energy scale from top panel. The plot shows how occupied the different states are throughout the free energy landscape, from blue (low occupancy) to deep red (high occupancy). The location of the unfolded and native basins are positioned ate similar Q and RMSD with similar occupation of states indicating a similar folding route for all three models.

Numerical simulations were performed with structured based model as described (see method section) (Table 1). The *top panel* in Figure 3 shows the free energy profile of 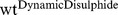 (black), 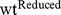 (blue) and 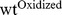 (red) respectively. The folding temperature T_f_, based on the free energy profile, was found for each model. Thus, all simulation were performed at their individual value of T_f_ (Table 1). The 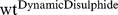 is a toy model exploring the free energy landscape with a structure based model, where the disulphide bond is treated as a typical native contact rather then a covalent bond. This is the model with the highest transition state (TS) value, found at Q = 0.4 and F(Q)/k_B_T = 6.6 (defined as Δf^+^), and with the highest cooperativity. These features appear where the largest changes are seen from the denatured state to the native state. The 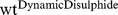 model explores the full cycle/route from a fully unfolded linear conformation to the compact native state. Representations of structures on the folding routes of the 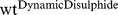 model are seen in Figure 3, *top panel*. Four structures are shown at two different values of Q (Q = 0.3, left side, and Q = 0.4, right side of Figure 3, *top panel*). Both reduced and oxidized assemblies are observed at the same Q vs. RMSD with the 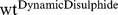 model. The highest TS values for both the 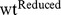 and 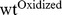 model are found at the same Δf^+^ (at Q = 0.4 and F(Q)/k_B_T = 6.6). The folded state ensemble resides at the same value of the reaction coordinates (at about Q = 0.8 and RMSD = 0.05) for the reduced and oxidized models, respectively. This indicates that the strength of the disulphide bridge does not influence the position of the native state. Despite these similarities, a noticeable difference is seen in the shape of F(Q) around TS. The 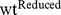 shows a more narrow TS with no significant traps on the folding route. The 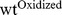 has a slightly broader TS, with a shoulder evident between N and the TS. A representation of a thermodynamic trajectory is given in Figure S2, comparing the 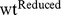 and 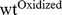 models. The oxidized route indicates several unsuccessful unfolding attempts, seen as a spike from the native state to the unfolded state. Given enough time or external support, this trapped state can be fully denatured. One might ask, why is the chain of leptin trapped in an unthreaded denatured state? (A) The 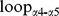 and the 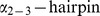 creates a topological obstacle which hinders the unthreading of the 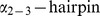 so that the denatured chain is trapped inside the 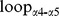. (B) There is one extra residue in the cut-off map than in the shadow map (P142, see methods). This residue is located in the bottom of the structure packing the threaded second loop tighter/closer to the C-terminus. (C) The trapped state has native contacts that stabilize the threaded state. Intramolecular contacts in α4 and α5 stabilize the 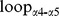 and decrease the effective size of the loop, thus trapping the 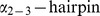. To investigate the density of states from a different perspective, the free energy is plotted as a function of Q and RMSD in the *bottom panel* of Figure 3. As expected, the RMSD of the unfolded basin is somewhat broader in the reduced state where the disulphide bridge is broken, as compared to the oxidized state with the fixed covalent 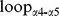. The oxidized route also shows a broader TS ensemble, probably due to the topological traps described previously. However the F(Q, RMSD) profiles reveal no significant differences between the folding routes.

**Table 1:** Parameters explaining the thermodynamic- and kinetic properties of the three models obtained from Cα simulations.

	Thermodynamic^a^		Kinetics^b^		
	*T*_f_ (k_B_T)	*F(Q)/K_B_T*	P_t_	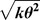	Unsuccessful events (%)
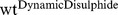	143	6.6	12626	47028	1
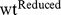	141	6.2	7151	9691	0
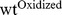	145	4.8	27346	54958	5

a Thermodynamic data is described by folding temperatures, T_f_, and the height of the free energy barrier, *F(Q)/K_B_T*.

b Kinetics data were fitted with the Gamma distribution 
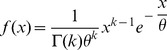
. The most probably time to fold P(t) is given by 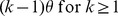 with the tail distribution given by 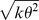. The last column represents the percentage of unsuccessful folding events, which are excluded from the Gamma distributions.

### Global Kinetic Analysis

**Figure 4:**
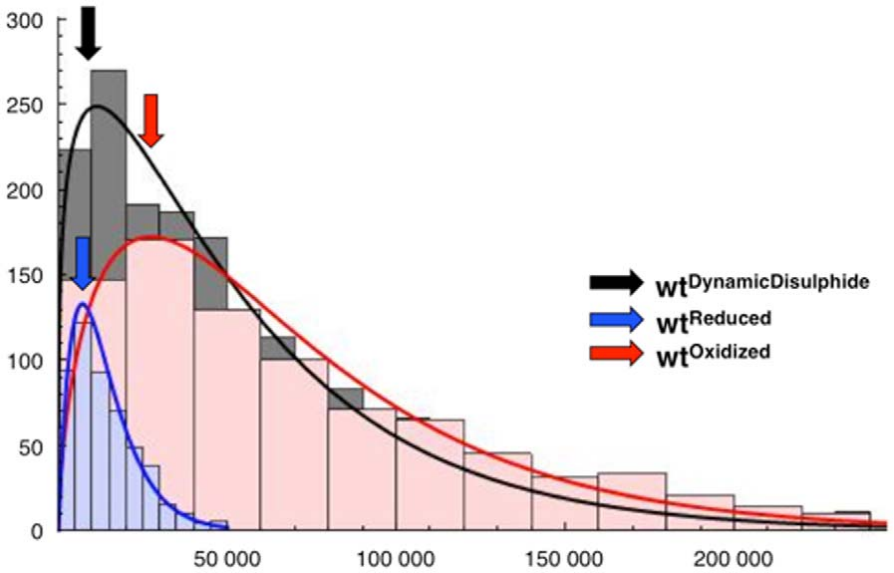
The distribution of the kinetic folding times described by a Gamma function [85], [86]. The fit is colored according to; 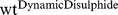 (black), 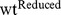 (blue) and 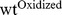 (Red). The most probable folding time, P_t_, (the position of the maximum of the fit) and the shape of the fit obtained for each model is shown in Table 1. P_t_ corresponds to the main folding route and is in the same range for the 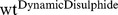 and 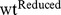 models. However, the tails of the distributions for these models are significantly different. In the case of the 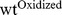 model P_t_ is three times bigger then for the 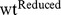 route and the tail distribution is similar to the 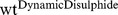.

In order to directly compare the kinetic folding, we performed simulations for our three models at the same temperature. Because the protein constructs have slightly different stabilities we compared them at 0.95T_f_ (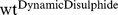), 0.96T_f_ (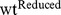) and 0.94T_f_ (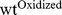), respectively (Figure 4). The number of successful folding event depends on the constraints introduced by the disulphide bridge formation. All routes in the unknotted protein (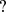) are successful, while a small number (1%) of the routes are trapped in the 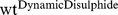 model, where the constraint is variable within the trajectories. The 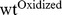 model has more traps with a 95% success rate. The distributions of kinetic folding times are best described by a Gamma distribution. For each fit we found the position of the maximum, which corresponds to the most probable time, P_t_ (Table 1). We found that P_t_ is in the same range for the 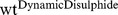 and 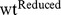 models, whereas P_t_ for 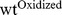 is significantly slower (at least two times slower). To characterise the difference in the time distributions we compared the shape of the Gamma distribution by calculating its variance 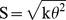 (Table 1). S is almost two times bigger, in the 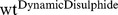 compared to the 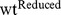 model, even though P_t_ is very similar. The discrepancy in S suggests a significant number of additional folding routes apart from the main route. This complexity is a consequence of the temporary constraints on the backbone introduced by the native contact between the two cysteines. This native contact imitates the formation of a disulphide bridge. Looking at the overall distribution of species over the entire folding reaction one observes a very long tail in the histogram of the 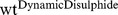 and 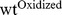 model. This indicates an additional complexity in the folding landscapes compared to the 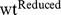 model. The 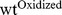 model shows an increase in P_t_, almost three and two times bigger then for the 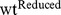 and the 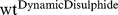 model respectively. This indicates a different main folding route, which is more complicated and thus slower. The longer folding times are a result of the more complicated topology in the oxidized state. However, the shape of the distribution is similar between the 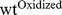 and the 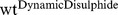 model. We found that S in the 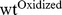 model is noticeably bigger than in 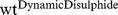 model (Table 1). The difference in S is a consequence of the constant constraints from the topological traps on the protein backbone introduced/implied by the disulphide bridge. If a more complex function is used to fit the data, an even more pronounced difference in the time distribution would be observed. All together, this shows that the folding route of the oxidized model are very complex even though the shape of the free energy F(Q, RMSD) is similar to the reduced model.

### Folding Mechanisms under Reduced and Oxidized Conditions is Conserved

**Figure 5:**
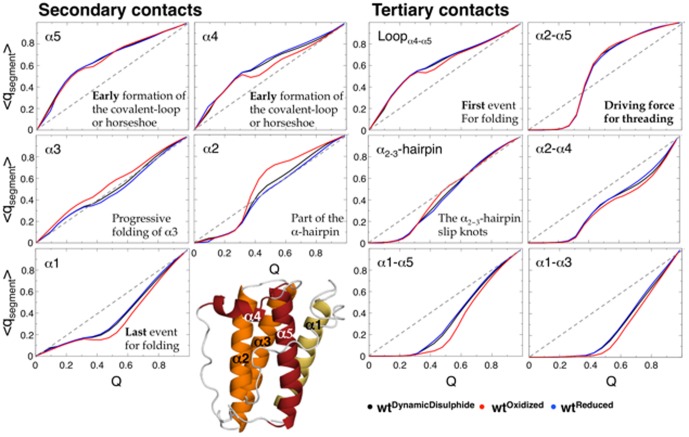
Probability of specific secondary structure elements and tertiary contacts on the folding routes. Characterizing the folding route based on the average contacts within specific secondary or tertiary contacts, 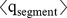, versus the nativeness of the overall fold, Q. *Left panel:* The plots show the distribution of internal secondary contacts for each α-helix versus Q. *Right panel:* The plots show the distribution of tertiary contacts between elements versus Q. Where 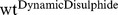, 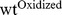 and 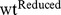 are shown in black, red and blue respectively. Progressive folding is indicated with a gray dotted line. The first element to fold is the covalent , followed by the 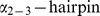. Finally, α1 is stabilized and folded to its correct position between α3 and α5 in the back of the 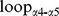. The structure of leptin is represented below the plots colored from red (early folding) to yellow (late folding).

Given the added topological constraint imposed by the disulphide bridge it was surprising that the folding landscapes of the oxidized and reduced proteins were largely similar. The question then arises, are the folding mechanisms for reduced and oxidized leptin truly conserved? In order to address this question we focus on the formation of specific secondary structure elements and helix-helix tertiary interactions 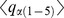 as a function of the total number of native contacts formed, Q (Figure 5). Each plot shows the increased formation of contacts for the three different models; 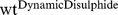 (black), 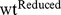 (blue) and 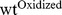 (red), at their respective folding temperatures, T_f_. Surprisingly, the folding routes appear conserved between the reduced and oxidized protein. The folding mechanism is always initiated by the formation of the 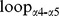 where more then 50% of the contacts are formed early (Q = 0.2). This finding indicates that the formation of the 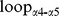 is independent of the oxidation state of disulphide bridge. Immediately following the formation of the 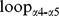 is the progressive folding of helix 3. Helix 2 starts to fold at Q>0.3 and 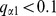, where more then half of the contacts in the 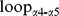 are formed. The N-terminal helix (α1) remains in a random conformation until both the 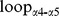 and 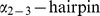 are formed (Q = 0.5 and 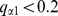). As α3 and α5 are formed in earlier events, where α1 is on the front side of the molecule, the last event for folding is to flip α1 to the back of the molecule, behind the 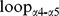.

It is important to note that in the absence of the disulfide bridge the open 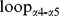, folds into a horseshoe conformation that brings the cysteines in close proximity. While the threading event discussed below technically only occurs in the oxidized protein, the same secondary and tertiary contacts are involved in the folding process with respect to the nearly closed conformation of the horseshoe in the reduced protein.

### Threading of the 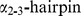 across the Closed 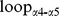


The oxidized state of leptin closes the 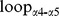 and introduces a slipknot event/motif in the folding landscape. Here, the 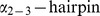 has to cross-over/thread-through the 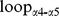 to reach the active native state (Figure 1). The main bottleneck in this case is due to the topological constraints introduced by the cysteine knotted conformation. The force to overcome this topological barrier is initiated from the formation of α2 and the contacts between α2–α3 and α2–α5 (Figure 5). Interestingly, the driving force for the threading event is also seen in the reduced state, where the formation of contacts between α2–α5 is conserved. Overall, the folding mechanism is conserved for both the reduced and oxidized protein. There are minor differences seen in the elements involved in the threading event in Figure 5. For example, enclosing the 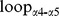 immobilizes α5 and places α3 in the correct slipknotted conformation, increasing the formation of α2 in the 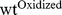 state. Also, folding in the oxidized state, with α4 packed within the covalent 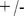, leads to a pause or stalling event in its formation. This data suggests that α4 cannot be well packed before the 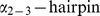 is threaded through the covalent 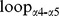. That is, too many constraints on the covalent 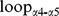 may impede the threading of the 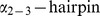 in the oxidized state.

### Exploration of the Folding Routes with an All-Atom Model

The Cα-model allows for a comprehensive description of the shape of free energy landscape. However, in the case of proteins with unique topologies, as seen in knotted proteins [23], [24], [28], the effect of the excluded volume may be significant as the internal radius of the slipknot is reduced and can impact the threading event. The effect of the excluded volume can also influence the unknotted state. These potential effects were explored with an All-Atom structure based model to explore the folding mechanism of leptin and the efficiency of threading when side groups are taken into account (for more details see the Supporting Information and Figure S3). We address whether the similarity between the three models seen from a Cα perspective is a result of the structure based models or whether the folding event in leptin is conserved even when steric crowding is imposed in the All-Atom model. While the free energy at the TS suggests a less cooperative folding event with higher levels of topological frustration in the All-Atom model, the observed folding mechanism in our models is conserved across all SBMs. 11

### *In vitro* Experiments

**Figure 6:**
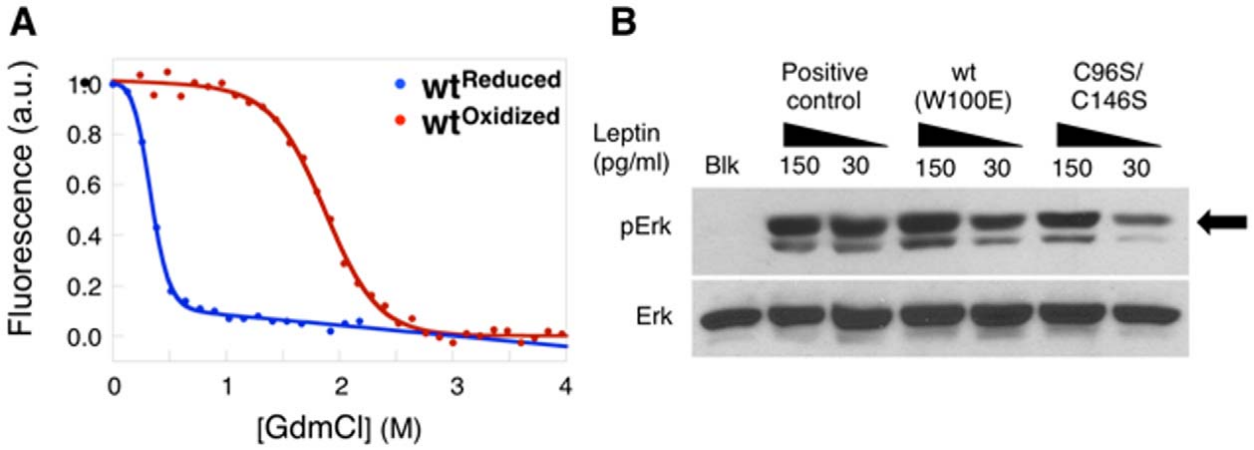
Experimental demonstration of the relative cooperativity of unfolding and signalling activity of WT and mutant leptin. *(A)* The equilibrium unfolding curves show the reduced (blue) and oxidized (red) states of leptin. The plots show that the oxidized protein is more stable then the reduced protein. *(B)* The activity assay was performed on human MCF-7 cell lines. We used a blank (Blk) to test the normal expression in these cell lines (Erk), a positive control (mouse leptin, purchased from Calbiochem) our purified oxidized leptin wt and our mutated leptin C96S/C146S, from left to right respectively. Stimulation results in the production of phosphorylated forms of Erk as indicated by the arrow (pErk).

Previously published *in vitro* experiments report different yields, expression levels and biological potencies of leptin renatured from *E. coli* inclusion bodies [56], [57], [58], [59], [60], [61], [62]. Our combined expertise in experimental folding [63], [64], [65], [66] and aggregation experiments [67], [68], [69] taken together with our results from SBMs allowed us to design a refolding protocol that not only limited protein aggregation but also afforded sufficient time for the threading event. The resulting refolded protein was characterized by optical as well as cellular activity assays. The equilibrium unfolding curves for the reduced (blue curve) and the oxidized (red curve) proteins are shown in Figure 6A. The data is fitted to a two-state model as described in [70] and is given in Table 2. The stabilizing effect of disulfide bridge formation is clearly evident in the shift of the mid-point (MP), as well as the change in the overall stability of the proteins in the oxidized and reduced curves. Surprisingly, the overall stability is very low compared to other helical-bundles (1.8^Reduced^ *versus* 3.4^Oxidized^ kcal/mol, respectively) [15], [16], [17], [18]. The m_D-N_ values are significantly different, i.e. the reduced state has an m_D-N_ value of 5.2 compared to 1.8 as seen for the oxidized state (Table 2). Clearly, a major contributor to this cooperativity change is due to the difference in solvent exposed surface area in the unfolded state, where the reduced protein unfolds to a linear protein while the oxidized protein is trapped in a denatured conformation where the covalent 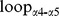 remains intact (Figure 1C).

**Table 2:** Experimental data from equilibrium curves of wt and mutated leptin.

	*m*_D-N_ a	MΠ^a^	ΔG_D-N_ a (kcal/mol)
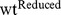	5.2	0.3	1.8
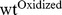	1.8	1.9	3.4

a All unfolding equilibrium data is fitted to a standard two-state equation (see method section).

To ensure that we refolded to the native state, we tested the activity of our purified protein with a cellular activity assay as described [71]. The results of these studies are shown in Figure 6B. The unphosphorylated kinase Erk (extracellular signal-regulated kinase) is constitutively produced in the absence of leptin stimulation. Previous studies suggest that the reduced form of leptin is not fully active [31], [42], [72]. Our activity assay reveals that the reduced form (mimicked by the mutations C96S/C146S) of leptin is capable of stimulating the Janus kinase/Signal transducer and activator of transcription (JAK/STAT) pathway, however the controlled dose-dependent assays indicate it is less active than the oxidized species. These results suggest that the reduced state has the correct conformation to bind the receptor and activate signaling cascade. The lower activity seen in the reduced state could be an effect of the shifted dynamics in the native state and/or an effect of the decrease in stability of the non cross-linked form of leptin. This could lead to either (A) a lower binding affinity (lower k_d_) or (B) a lower signaling cascade when bound to the receptor, because binding and signaling could be decoupled [73], [74]. However, when the leptin receptor JAK/STAT signaling cascade is initiated by stimulation with quality controlled leptin (purchased from Calbiochem) or our purified oxidized protein we observe identical phosphorylation levels of pErk. Our simulations of the native state dynamics reveals that the disulphide bond functions as a point of tension that influences local motions on distal receptor-binding sites. Breaking the disulphide bonds increases the local motions in the bottom of the structure, which binds to the receptor complex. The dynamics of the top part of the structure increases when the disulphide bridge is formed, indicating that the disulphide bridge might function as a tension point. Moreover, increased dynamics of the protein could enhance receptor binding/signaling through induced fit mechanisms.

## Conclusion and Discussion

**Figure 7:**
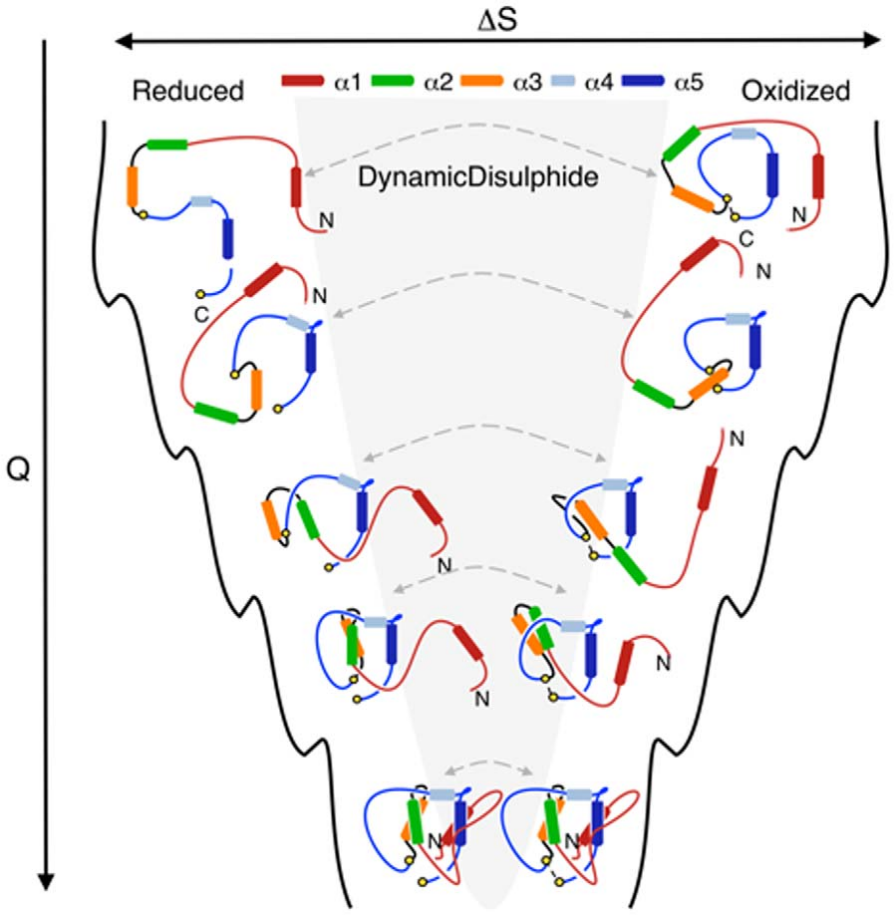
Cartoon representation of the folding landscape of leptin. All possible geometries are observed theoretically, however, the route indicated by the grey shadow is not accessible through our experiments. The conversions throughout the folding landscape are as follows: The top of the figure shows the denatured states; with high entropical freedom and small numbers of Q. As the protein folds several similar states reduced/oxidized can be seen at the same level of Q, indicating that the folding routes are very similar between the two forms of leptin. Both the reduced and oxidized route leads to a correctly folded native state.

We found that leptin possesses a unique cysteine knotted topology. We investigated the impact and importance of the knotted topology on the correct folding of leptin as well as its correlation with receptor binding and activity. Our results show that the folding mechanism appears to be very similar for the unknotted (reduced) and knotted (oxidized) forms of leptin (Figure 7). Therefore, topological constraints imposed by the disulphide bridge do not determine the overall shape of the free energy landscape associated with the folding event. However, the disruption of the disulphide bond plays a central role [2], [3], [4] in efficient folding by increasing the folding rate. This suggests a new interpretation of previously published experimental results [5], [6]. Misfolding of leptin is not due to the failure of forming the correct disulphide bridge, rather it is a complication of mutations altering the unique folding route associated with a slipknotted topology. The experimentally observed unsuccessful routes for the reduced form are probably a consequence of the harsh conditions used in previous studies [29], [30], [33]. While the current view is that disease-associated mutations in leptin aggregate as a result of incorrect disulphide bridge formation [2], [4], [5], [6], we propose a new interpretation. Our work indicates that the driving force to thread the 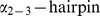 across the 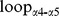 comes from network of native contacts between helix 2 and helix 5 (Figures 1, 4). We can also demonstrate that the threading event occurs both in the reduced and oxidized state. Based on this data we suggest that these mutations in leptin structure can play three roles: (A) Disruption of native interactions which are responsible for dragging the 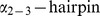, (B) Decrease of the effective size of the covalent 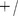 across which the 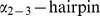 has to be threaded, and (C) Mutations can also affect the receptor binding sites, thus impair or block receptor binding [13], [75], [76]. The remaining question then is to explore the threading event and folding of leptin through a more detailed study of different point mutation within the slipknotted topology. Our work also suggest that the disulphide bridge plays a more important regulatory role then expected, where the novel landscape allows for a subtle, yet robust mechanism where a singly disulphide bridge is able to modulate distal regions needed for receptor binding, which has not been observed previously. This unique mechanism appears to mediate biological function that has yet to be observed. Only the combination of theoretical studies and *in vitro* experiments allowed us to discover this new folding mechanism, which was corroborated with assays and confirms this new important finding. Such an understanding opens many new possibilities for exploring leptin function and further development of therapeutics. Inhibition of leptin signaling is beneficial in models of fibrosis and inflammation while leptin activation strategies are beneficial in the treatment of cachexia and anorexia [74].

## Methods

### Structure Based Models (SBM)

Our models are based on the hypothesis that pairs of interacting residues (*i,j*) in the native state of the protein provide, on average, more stability throughout the folding process than non-native contacts [54], [77]. This implies that the protein is minimally frustrated [52], [53], [55] and provides the basic framework to construct SBM [52], [53]. In this work we investigate the thermodynamics and kinetics of folding using both Cα [51], [78] and All-Atom [51], [53] SBMs.

### The Cα Model

In the Cα model for leptin each amino acid is represented as a single bead at the Cα position. The interacting pairs, amino acids *i* and *j* in the native state, are identified based on a shadow map (Figure S4) [79], [80]. The basic form of the potential is,
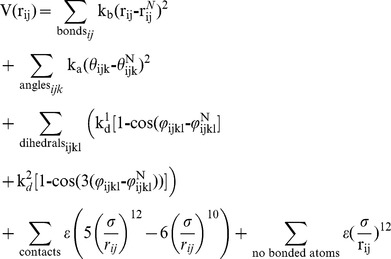
where the last two terms correspond respectively to attractive and repulsive interactions. 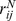 denotes the native distance between atoms *i* and *j* along the sequence. The topology of the chain is described by the native angle 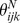 between the bonds connecting atom pairs *ij* and *jk*, and the native dihedrals 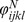 describe the angle between the planes defined by atoms *ijk* and *jkl*. The strength of the interactions are described by the reduced energy units k_b_ = 2×10^4^ ε/nm^2^, k_a_ = 40 ε/rad^2^, k^1^_d_ = ε and k^2^_d_ = 0.5ε. The details of the model are characterised elsewhere [51], [52], [53], [78].

### The All-Atom Model

In the All-Atom (AA) model all heavy atoms are taken into account [51], [53]. Here, we found that a 5 Å cut-off map represents the features of leptin is optimal for characterization of leptin with the current model (Supporting Information and Figure S4). The potential for the All-Atom model is an extension of the Cα model. However, additionally it takes into account all heavy atoms. Thus, two additional terms are added to maintain the conformation of the backbone and amino acid side chains. The details of the All-Atom model are presented elsewhere [51], [53].

Our models [51], [53] are parameterized based on empirical values given from the protein structure (PDB code 1AX8 [14]). The available crystal structure does not describe the coordinates of residues 25–38 in loop 1 and were reconstructed with the server [81]. The results of the current studies were independent of the loop conformation.

### Simulations and Data analysis

The web server (http://smog.ucsd.edu) [80] was used to create input files to perform simulations with the GROMACS 4.0.5 software package [82] (Figure S4). All results are presented with reduced units and the integration steps, t = 0.0005 was used throughout. Several constant temperature runs, including transitions from fully unfolded to folded native states, were performed and combined with the Weighted Histogram Analysis Method (WHAM) [83], [84] to create free energy profiles (F(Q)). The apparent folding temperatures are estimated from each maximum peak in each specific heat curve. For a formed native contact the energy gain is measured by epsilon (*ε*), and thus the temperatures and energies reported in this paper are measured in units of *ε*. In the case of the reduced model we used an *ε* of 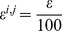, which respectively will rescale the shape of the potential and weakening the contact. To evaluate the nativeness of a structure we used Q, the number/fraction of native contacts within a structure [51]. A contact between the native pair ij is considered formed if it satisfies r_ij_<γr^ij^_0_, where γ≈1.2–1.4 and r^ij^_0_ describe native distance between ij. Additionally, we also characterize the complexity introduced from the cysteine knotted structure with the Root-Mean-Square Distance (RMSD) of the configuration from the native state.

### The 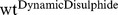, 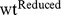 and 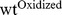 States of Leptin

**Figure 8:**
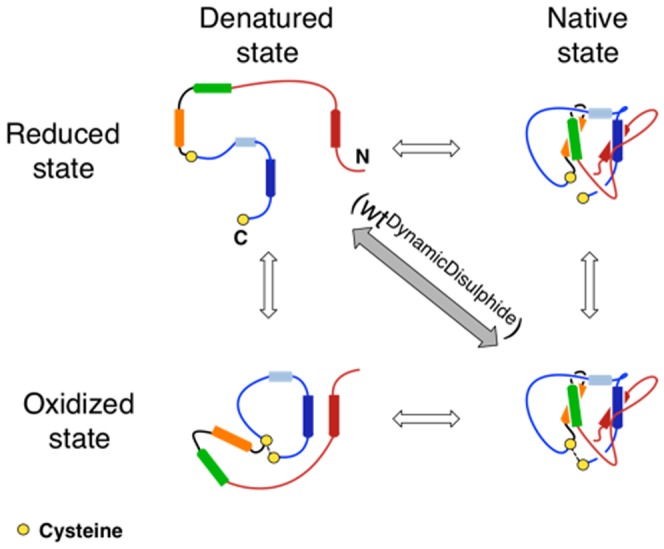
Cartoon of the different folded/unfolded states. The cartoon represents reduced and oxidized leptin, seen in the *top-* and *bottom panel* respectively. The *left-* and *right panel* shows the denatured and native state respectively. The grey colour indicates the interconversion between two constructs as a result of the numerical simulation where the disulphide bond is continuously made and broken during simulation (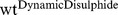). Spontaneous reduction of the protein (grey pathway) is not observed experimentally.

Leptin has a continuum of states from the oxidized native to the reduced unfolded forms. For this reason, we constructed three SBMs to model different physical/chemical conditions for leptin described below (Figure 8).

### The DynamicDisulphide model, 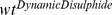 (Figure 8, grey arrow)

This model corresponds to a condition where the contacts between the two cysteines spontaneously is made and broken along the folding/unfolding route. In this case, the folding mechanism cannot be observed experimentally. However it represents an ideal *toy model* for theoretical investigation. The spontaneous formation of contact between the cysteines is modeled by making the disulfide bond a Lenard Jones type of contact equivalent to the other contacts (*ε* ^C96–C146^ = *ε* = *constant*).

### The Reduced model, 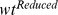 (Figure 8, top panel)

This model corresponds to the conditions where the disulphide bridge cannot form, mimicking the strongly reducing conditions applied experimentally. The strength of the disulphide bond was significantly decreased in this SBM 
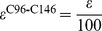
. The strength of native contacts in the vicinity of disulphide bridge were also decreased to insure that there are no contributions from the nearest neighbour. Contacts between residue *i* and *j*: F92–D141, F92–L142, F92–S143, F92–P144, S95–P144 and C96–P144 are represented with 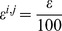.

Furthermore, to test this reduced SBM we also used a construct where only the strength of the disulphide bond was reduced, 
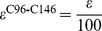
. The result agrees with the data from the reduced model and additionally shows slightly broader diversity in folding pathway as expected (data not shown).

### The Oxidized model, 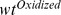 (Figure 8, bottom panel)

This model corresponds to a condition where the disulphide bridge is always intact, and mimics the oxidized conditions applied experimentally. In this SBM the strength of disulphide bridge was modelled as strong as a peptide bond, as 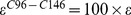.

### Native state dynamics

To characterize the native state motions we performed All-Atom simulations of both the oxidized and reduced form of leptin. We deleted the first 500 frames of each trajectory to ensure that the system was equilibrated. Based on the obtained trajectories we calculate and diagonalize the (mass-weighted) covariance matrix for the backbone of the protein using GROMACS standardized tools. All structures are fitted to the native state of leptin available in PDB (1AX8 [14]). We obtained a full set of eigenvectors from which we analyze the first four. These vectors describe the slowest motions of the protein. Next we calculate the principal components by making the projections of the trajectory on the eigenvectors. To characterize the amplitude of the motion of each atom in respect to the native structure we calculated the root mean square deviation based on the first, second, third and fourth eigenvectors.

### Kinetics Analyses

The refolding times from 500–800 trajectories where analyzed for all three models. A Gamma distribution function describes the distributions of folding times in the best way [85], [86]. The functions were fitted with Mathematica 8.5, to optimize the observed histogram distribution.

### Robustness of the SBM

We tested several SBMs to explore the robustness of the observed mechanisms. Different attractive and repulsive potentials (Gaussian potential [87], Lennard Jones potential [88], 10/12 potential [88]) and different contacts maps were investigated (cut-off map with different cut-off values and shadow map [79], [80] (Figure S4). We found that the folding and unfolding mechanisms do not depend on the details of the SBM employed.

### Protein Expression and Purification

The leptin gene (purchased from GenScript USA Inc, Piscataway) was cloned into a pET-3A vector with restriction sites NdeI and BamHI and transformed into competent *E. coli* strain BL21 (DE3) expression cells. One point mutation was introduced at position 100 (W100E), to prevent precipitation [1]. Mutations were performed with the Quick-Change site-directed mutagenesis kit (Stratagene), and oligonucleotides were purchased from Integrated DNA technology. The mutations and integrity of the amino acid sequence were confirmed by sequencing of the entire gene (ETON biosciences). The protein was over expressed and purified from inclusion bodies [56]. Unfolded protein was loaded onto a gel filtration (Sephacryl S-200) column, refolded and loaded onto an S-200 column again to prevent formation of oligomeric species. Oxidized and reduced glutathione was used as shuffling reagent to insure correct formation of the disulphide bridge. The purity of the protein was confirmed by SDS-page and the identity by mass spectrometry.

### Activity Assay

The human breast cancer MCF-7 cell line was used to investigate the biologic activity of our purified wild-type (wt) and mutated leptin. The cell lines were cultured in DMEM (Dulbecco's modified Eagle's medium) with 10% fetal bovine serum. The MCF-7 cells were starved overnight without serum and subsequently stimulated for 20 minutes by mouse leptin (Calbiochem), wt human leptin or mutated human leptin (C96S/C146S), respectively. Immunoblotting of cell lysates were used to perform activity assays with the antibodies pErk (phosphorylated extracellular signal-regulated kinase, cell signaling), and Erk (extracellular signal-regulated kinase, cell signaling) [71].

Mouse leptin (positive control, purchased from Calbiochem EMD), purified human wt and mutated leptin were used at different concentrations to treat MCF-7 cells containing the leptin receptor. As a control, the rat neuronal cell line PC12^LeRb^ was also infected with leptin indicating the same results as seen with the human cells (result not shown) [89].

### Native State Stability analyses

Equilibrium unfolding titrations were measured using average fluorescence wavelength [90]. The single tryptophan residue 138 is a useful probe of the global unfolding reaction. Fluorescence spectra were collected with an excitation of 280 nm and emission collected from 300–450 nm, both for reduced and oxidized leptin. Protein samples were prepared at a concentration of 12 µM in a buffer solution (BisTris 10 mM at pH 6.3) containing varying concentrations of denaturant ranging from 0 to 4 M GdmCl. The fluorescence data were collected at an emission wavelength between 300–450. The protein was incubated for 6 hours with 20 mM of TCEP (tris(2-carboxyethyl)phosphine) to reduce the disulphide bridge, a time sufficient to break the disulphide according our iodoacetic acid assay results (data not shown) [91], [92], [93]. Leptin shows a hyperfluorescence behavior/signal. Therefore, the linear regime of the equilibrium curves was fitted to the standard two-state equation as described for [70]

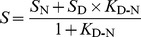
where
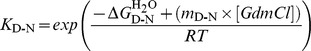
where m_D-N_ is the linear dependence of ΔG_D-N_ on denaturant concentration and 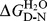 is the free energy of unfolding at 0 M GdmCl. S is the signal of the native and denatured states.

## Supporting Information

Figure S1 **Sequence alignment and structural mapping of leptin homologs.** (A)The amino acid alignment of human leptin and six of its homologs [14], [41], [42]. The sequence conservation between the different species 38–86% similar to human leptin. Even though they are different, all of them have two cysteines where one of them is the N-terminal residue and the other is positioned close to helix 3. (B) The wild type leptin structure and the backbone of predicted tertiary structures of mouse, rat, frog and puffer fish leptin (SWISS-MODEL automated protein homology-modelling server where the structures are based on human leptin [94], [95], [96]).
(TIFF)

Figure S2
**Evidence for the denatured threaded state prior to full unfolding.** The trajectory shows the energy levels of the folded- (around 0) and unfolded basins (around 250) for 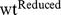 (black ) and 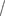 (red) respectively. The plot also indicate that there are several unsuccessful unfolding attempts on the 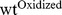 route from N to U. The unfolded chain is trapped inside 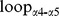 (helix 2 and 3 shown in white in the structure B), leading to a denatured threaded state. This represents the typical subpopulation during the unfolding of leptin. The structures show the different states, i.e. the native state (A), trapped threaded state (B) and the unfolded unthreaded state (C), from the folding routes in an All-Atom representation.(TIFF)

Figure S3
**The All-Atom simulations of leptin.** The free energy landscape F(Q, RMSD) together with the free energy plot of the All-Atom simulation (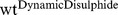). The plots show that there are no significant shifts of the denatured and native basins. As appose to the broad TS seen in the oxidized state in Figure 3, we see the potential of an intermediate formation in the All-Atom model.(TIFF)

Figure S4 **Contact maps for leptin showing the probability of contact formation at the TS.** The contact map is shown at the TS (Q = 0.4). Leptin displays a diffuse TS where all helices, except α1, are involved. They also indicate/show that the TS is very similar between the different Cα- versus the SBMs.
(TIFF)

Table S1 **Kinetics data.**
(DOC)

Table S2 **Thermodynamics.**
(DOC)
