# Supplementary figures and images for: The Unique Cysteine Knot Regulates the Pleotropic Hormone Leptin
Source: PLoS One. 2012 Sep 24;7(9):e45654. doi: 10.1371/journal.pone.0045654 (PMC3454405; doi:10.1371/journal.pone.0045654)

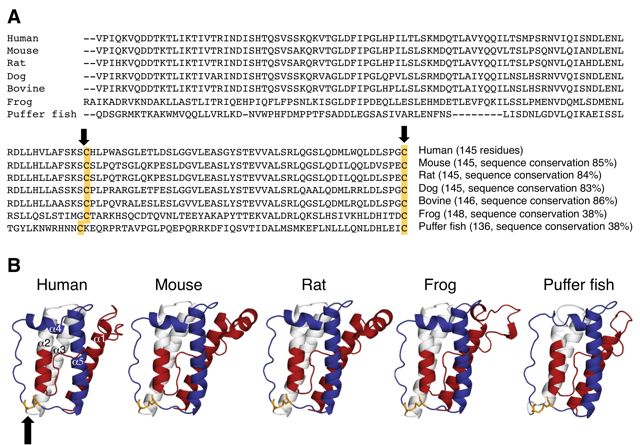

Supplement: Figure S1 — Sequence alignment and structural mapping of leptin homologs. (A)The amino acid alignment of human leptin and six of its homologs [14], [41], [42]. The sequence conservation between the different species 38–86% similar to human leptin. Even though they are different, all of them have two cysteines where one of them is the N-terminal residue and the other is positioned close to helix 3. (B) The wild type leptin structure and the backbone of predicted tertiary structures of mouse, rat, frog and puffer fish leptin (SWISS-MODEL automated protein homology-modelling server where the structures are based on human leptin [94], [95], [96]). (TIFF) [file pone.0045654.s001.tif]

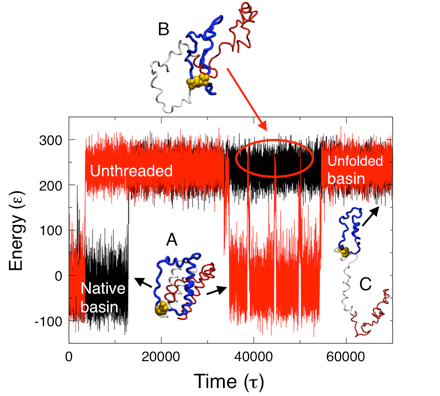

Supplement: Figure S2 — Evidence for the denatured threaded state prior to full unfolding. The trajectory shows the energy levels of the folded- (around 0) and unfolded basins (around 250) for (black ) and (red) respectively. The plot also indicate that there are several unsuccessful unfolding attempts on the route from N to U. The unfolded chain is trapped inside (helix 2 and 3 shown in white in the structure B), leading to a denatured threaded state. This represents the typical subpopulation during the unfolding of leptin. The structures show the different states, i.e. the native state (A), trapped threaded state (B) and the unfolded unthreaded state (C), from the folding routes in an All-Atom representation. (TIFF) [file pone.0045654.s002.tif]

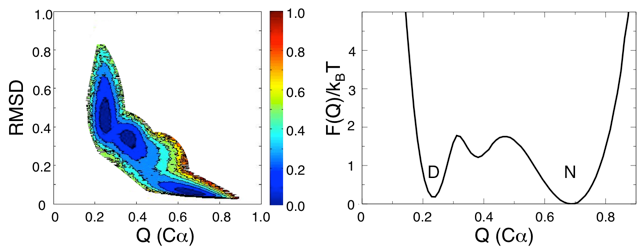

Supplement: Figure S3 — The All-Atom simulations of leptin. The free energy landscape F(Q, RMSD) together with the free energy plot of the All-Atom simulation (). The plots show that there are no significant shifts of the denatured and native basins. As appose to the broad TS seen in the oxidized state in Figure 3, we see the potential of an intermediate formation in the All-Atom model. (TIFF) [file pone.0045654.s003.tif]

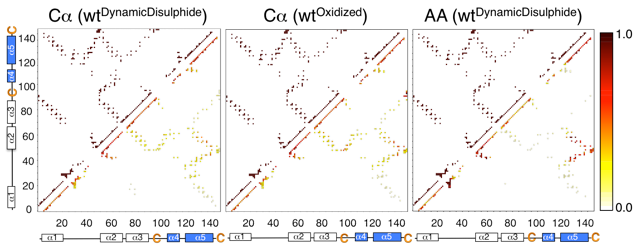

Supplement: Figure S4 — Contact maps for leptin showing the probability of contact formation at the TS. The contact map is shown at the TS (Q = 0.4). Leptin displays a diffuse TS where all helices, except α1, are involved. They also indicate/show that the TS is very similar between the different Cα- versus the SBMs. (TIFF) [file pone.0045654.s004.tif]
