# Supplementary material for: The Unique Cysteine Knot Regulates the Pleotropic Hormone Leptin
Source: PLoS One. 2012 Sep 24;7(9):e45654. doi: 10.1371/journal.pone.0045654 (PMC3454405; doi:10.1371/journal.pone.0045654)
Supplement: Table S2 — Thermodynamics. (DOC) [file pone.0045654.s006.doc]

Table S2. Thermodynamics.

|  | *T*ime steps | *Frames* | Trajectories |
| --- | --- | --- | --- |
| $wt^{DynamicDisulphide}$ | 60 | 50000 | 61 |
| $wt^{Reduced}$ | 60 | 50000 | 61 |
| $wt^{Oxidized}$ | 60 | 50000 | 61 |
